# Supplementary material for: Drug repurposing for aging research using model organisms
Source: Aging Cell. 2017 Jun 16;16(5):1006–15. doi: 10.1111/acel.12626 (PMC5595691; doi:10.1111/acel.12626)
Supplement: Supplementary file 7 — Data S1 Zip‐Archive of all report cards. [file ACEL-16-1006-s007.zip › RC_EVP.pdf]

EVP

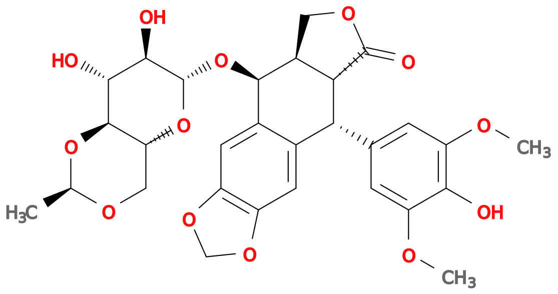

Database identifiers

ChEMBLCompound	CHEMBL44657
DrugBank	DB00773
CHEBI	4911
ZINC	ZINC03938684
eMolecules	32278021
eMolecules	537894

Ranking

	Rank	Score
Drosophila	524/697	0.232
C. elegans	418/591	0.127

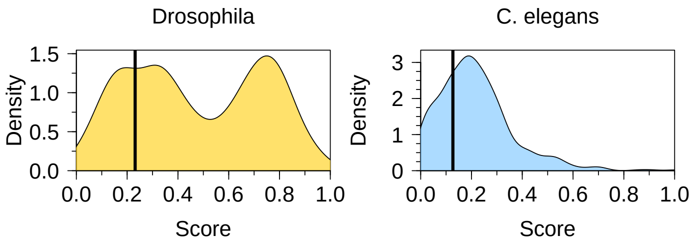

	Ageing implication	Domain conservation	Binding site conservation	Binding affinity	Bioavailability	Lipinski	Promiscuity	Purchasability	Drug approval	**Total**
Drosophila	0.624	0.963	0.777	0.314	(0.9)	-0.1	-0.0	0.1	0.1	0.232
C. elegans	0.624	0.963	0.721	0.314	0.201	-0.1	-0.0	0.1	0.1	0.127

Names

- **etoposide**
- 4-demethylepipodophyllotoxin beta-D-ethylideneglucoside
- Toposar
- trans-Etoposide
- Vepesid
- VP-16

Roles

antineoplastic agent, DNA synthesis inhibitor, DNA topoisomerase II inhibitor

Status

Approved drug (according to ChEMBL)	Yes
Administration Route	Oral, Intravenous
Number of Rule of 5 violations	2
Binding affinity to original target in log units (RF-Score prediction)	4.22
Burns *C. elegans* bioavailability prediction	-11.84

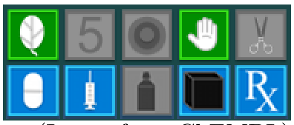

(Image from ChEMBL)

Medical Information

Indicatation: For use in combination with other chemotherapeutic agents in the treatment of refractory testicular tumors and as first line treatment in patients with small cell lung cancer. Also used to treat other malignancies such as lymphoma, non-lymphocytic leukemia, and glioblastoma multiforme.
Mechanism of action: Etoposide inhibits DNA topoisomerase II, thereby inhibiting DNA religation. This causes critical errors in DNA synthesis at the premitotic stage of cell division and can lead to apoptosis of the cancer cell. Etoposide is cell cycle dependent and phase specific, affecting mainly the S and G2 phases of cell division. Inhibition of the topoisomerase II alpha isoform results in the anti-tumour activity of etoposide. The drug is also capable of inhibiting the beta isoform but inhibition of this target is not associated with the anti-tumour activity. It is instead associated with the carcinogenic effect.
Toxicity: Side effects include alopecia, constipation, diarrhea, nausea and vomiting and secondary malignancies (leukemia).
Metabolism: Primarily hepatic (through O-demethylation via the CYP450 3A4 isoenzyme pathway) with 40% excreted unchanged in the urine. Etoposide also undergoes glutathione and glucuronide conjugation which are catalyzed by GSTT1/GSTP1 and UGT1A1, respectively. Prostaglandin synthases are also responsible for the conversion of etoposide to O-demethylated metabolites (quinone).
Retail: APP Pharmaceuticals, Baxter International Inc., Bedford Labs, Ben Venue Laboratories Inc., Bristol-Myers Squibb Co., Hospira Inc. and 12 more suppliers
Toposar 500 mg/25 ml vial - 1.06 USD / ml
Toposar 1000 mg/50 ml vial - 1.12 USD / ml
Toposar 100 mg/5 ml vial - 2.25 USD / ml
Etoposide 100 mg/5 ml vial - 28.89 USD / ml
Etoposide 50 mg capsule - 47.64 USD / capsule
Etopophos 100 mg vial - 159.55 USD / vial
and 2 more options

(Information from DrugBank)

Compound Target Characteristics

DNA topoisomerase 2-beta

Best gene implication in ageing for this target family came from gene F1M7A0 via mapping the annotation from RGD 62048 annotated in RGD 2014-03-11. Annotation GO 7568 (aging) was Inferred from Expression Pattern

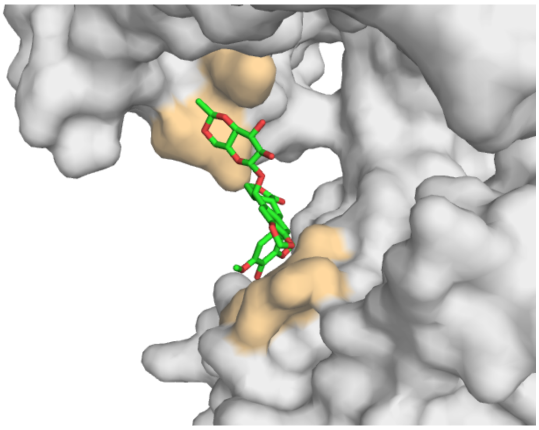

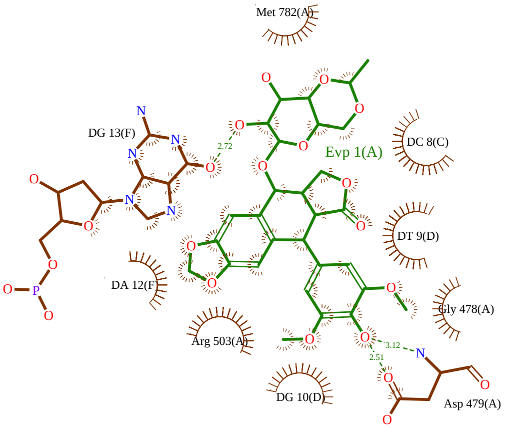

protein	amino acids contacts (binding site)				
PDB:3qx3:chainA:Q02880	G	D	R	Q	M
tr:E9PCY5:E9PCY5_HUMAN	G	D	R	Q	M
sp:Q02880:TOP2B_HUMAN	G	D	R	Q	M
tr:F1M7A0:F1M7A0_RAT	G	D	R	M	M
sp:Q64511:TOP2B_MOUSE	G	D	R	Q	M
tr:Q6NR40:Q6NR40_DROME	G	D	R	V	M
sp:P15348:TOP2_DROME	G	D	R	V	M
tr:G5ECQ8:G5ECQ8_CAEEL	G	D	R	Q	G
sp:P34534:TOP2M_CAEEL	G	D	K	E	R
sp:Q23670:TOP2_CAEEL	G	D	R	Q	G
tr:E9P8H0:E9P8H0_YEASX	G	D	R	Q	Q
tr:E9P8H2:E9P8H2_YEASX	G	D	R	Q	Q
tr:E9P8H4:E9P8H4_YEASX	G	D	R	Q	Q
tr:E9P8H5:E9P8H5_YEASX	G	D	R	Q	Q
tr:E9P8H7:E9P8H7_YEASX	G	D	R	Q	Q
tr:E9P8H8:E9P8H8_YEASX	G	D	R	Q	Q
tr:Q7LMA0:Q7LMA0_YEASX	G	D	R	Q	Q
tr:E9P8G9:E9P8G9_YEASX	G	D	R	Q	Q
sp:P06786:TOP2_YEAST	G	D	R	Q	Q

	whole protein		domain-based		contact-based	
protein	ident	simil	ident	simil	ident	simil
PDB:3qx3:chainA:Q02880	1.0	1.0	1.0	1.0	1.0	1.0
tr:E9PCY5:E9PCY5_HUMAN	0.71	0.71	1.0	1.0	1.0	1.0
sp:Q02880:TOP2B_HUMAN	1.0	1.0	1.0	1.0	1.0	1.0
tr:F1M7A0:F1M7A0_RAT	0.64	0.83	0.87	0.96	0.8	0.77
sp:Q64511:TOP2B_MOUSE	0.96	0.98	0.99	1.0	1.0	1.0
tr:Q6NR40:Q6NR40_DROME	0.41	0.58	0.75	0.93	0.8	0.78
sp:P15348:TOP2_DROME	0.47	0.71	0.75	0.93	0.8	0.78
tr:G5ECQ8:G5ECQ8_CAEEL	0.25	0.42	0.66	0.9	0.8	0.7
sp:P34534:TOP2M_CAEEL	0.29	0.55	0.51	0.83	0.4	0.72
sp:Q23670:TOP2_CAEEL	0.45	0.73	0.73	0.92	0.8	0.7
tr:E9P8H0:E9P8H0_YEASX	0.34	0.64	0.58	0.86	0.8	0.77
tr:E9P8H2:E9P8H2_YEASX	0.34	0.64	0.58	0.86	0.8	0.77
tr:E9P8H4:E9P8H4_YEASX	0.34	0.64	0.58	0.86	0.8	0.77
tr:E9P8H5:E9P8H5_YEASX	0.34	0.64	0.58	0.86	0.8	0.77
tr:E9P8H7:E9P8H7_YEASX	0.34	0.64	0.58	0.86	0.8	0.77
tr:E9P8H8:E9P8H8_YEASX	0.34	0.64	0.58	0.86	0.8	0.77
tr:Q7LMA0:Q7LMA0_YEASX	0.34	0.64	0.58	0.86	0.8	0.77
tr:E9P8G9:E9P8G9_YEASX	0.34	0.64	0.58	0.86	0.8	0.77
sp:P06786:TOP2_YEAST	0.34	0.64	0.58	0.86	0.8	0.77

Top2 (FBgn0003732) associated phenotypes

developmental rate defective, heat sensitive, lethal - all die before end of first instar larval stage, lethal - all die before end of second instar larval stage, lethal - all die during first instar larval stage,

male fertile, neuroanatomy defective, partially lethal - majority die, small body, somatic clone

(Information from FlyBase)

Top2 (UniProt:Q6NR40) annotation
Function: Control of topological states of DNA by transient breakage and subsequent rejoining of DNA strands. Topoisomerase II makes double-strand breaks. (RuleBase:RU000381).
Subunit: Homodimer. ECO:0000256—RuleBase:RU000381.

(Information from UniProt)

Top2 (UniProt:P15348) annotation
Function: Control of topological states of DNA by transient breakage and subsequent rejoining of DNA strands. Topoisomerase II makes double-strand breaks.
Cofactor: Mg(2+) Evidence=(PROSITE-ProRule:PRU00995); Name=Mn(2+); Xref=ChEBI:CHEBI:29035; Evidence=(PROSITE-ProRule:PRU00995); Name=Ca(2+); Xref=ChEBI:CHEBI:29108; Evidence=(PROSITE-ProRule:PRU00995); Note=Binds two Mg(2+) per subunit. The magnesium ions form salt bridges with both the protein and the DNA. Can also accept other divalent metal cations, such as Mn(2+) or Ca(2+). (PROSITE-ProRule:PRU00995);
Subunit: Homodimer.
Subcellular location: Nucleus.
Miscellaneous: Eukaryotic topoisomerase I and II can relax both negative and positive supercoils, whereas prokaryotic enzymes relax only negative supercoils.

(Information from UniProt)

cin-4 (WBGene00022854) associated phenotypes
embryonic lethal, mitotic chromosome segregation variant

(Information from WormBase)

R05D3.12 (UniProt:P34534) annotation
Function: Control of topological states of DNA by transient breakage and subsequent rejoining of DNA strands. Topoisomerase II makes double-strand breaks (By similarity).
Cofactor: Mg(2+) Evidence=(PROSITE-ProRule:PRU00995); Name=Mn(2+); Xref=ChEBI:CHEBI:29035; Evidence=(PROSITE-ProRule:PRU00995); Name=Ca(2+); Xref=ChEBI:CHEBI:29108; Evidence=(PROSITE-ProRule:PRU00995); Note=Binds two Mg(2+) per subunit. The magnesium ions form salt bridges with both the protein and the DNA. Can also accept other divalent metal cations, such as Mn(2+) or Ca(2+). (PROSITE-ProRule:PRU00995);
Subunit: Homodimer.
Subcellular location: Mitochondrion

(Information from UniProt)

K12D12.1 (UniProt:Q23670) annotation
Function: Control of topological states of DNA by transient breakage and subsequent rejoining of DNA strands. Topoisomerase II makes double-strand breaks (By similarity).
Cofactor: Mg(2+) Evidence=(PROSITE-ProRule:PRU00995); Name=Mn(2+); Xref=ChEBI:CHEBI:29035; Evidence=(PROSITE-ProRule:PRU00995); Name=Ca(2+); Xref=ChEBI:CHEBI:29108; Evidence=(PROSITE-ProRule:PRU00995); Note=Binds two Mg(2+) per subunit. The magnesium ions form salt bridges with both the protein and the DNA. Can also accept other divalent metal cations, such as Mn(2+) or Ca(2+). (PROSITE-ProRule:PRU00995);
Subunit: Homodimer.
Subcellular location: Nucleus
Miscellaneous: Eukaryotic topoisomerase I and II can relax both negative and positive supercoils, whereas prokaryotic enzymes relax only negative supercoils.

(Information from UniProt)
